# Prospective Observational Study Investigating the Association of Fetal Distress and Oligohydramnios With Antenatal Ultrasonographic Umbilical Cord Hypercoiling

**DOI:** 10.7759/cureus.107944

**Published:** 2026-04-29

**Authors:** Prateeti Baruah, Subrat Panda, Ananya Das, Nalini Sharma, Amrita Datta, Rituparna Das, Donboklang Lynser, Himangshu Malakar, Dibyajyoti Saikia, Indrani Sarma

**Affiliations:** 1 Obstetrics and Gynecology, All India Institute of Medical Sciences, Guwahati, IND; 2 Obstetrics and Gynecology, North Eastern Indira Gandhi Regional Institute of Health and Medical Sciences (NEIGRIHMS), Shillong, IND; 3 Obstetrics and Gynecology, All India Institute of Medical Sciences, Kalyani, IND; 4 Radiodiagnosis, North Eastern Indira Gandhi Regional Institute of Health and Medical Sciences (NEIGRIHMS), Shillong, IND; 5 Pharmacology, All India Institute of Medical Sciences, Guwahati, IND

**Keywords:** antenatal complications, antenatal findings, assessment, logistic regression, multivariate models, umbilical cord coiling

## Abstract

Background: While abnormal umbilical cord coiling has been linked to various adverse perinatal outcomes, the antenatal associations remain poorly characterized and inconsistently reported across studies.

Objective: We sought to evaluate whether ultrasonography-derived umbilical cord coiling parameters, particularly hypercoiling, are associated with fetal distress and oligohydramnios in singleton pregnancies.

Methods: We conducted an observational analysis of 123 singleton pregnancies with complete follow-up data. Hypercoiling was defined using a prespecified coil-density cutoff of >0.3 coils/cm. We performed univariate logistic regression for each outcome; variables were based on clinical relevance, followed by prespecified multivariable models that included maternal age, gravida, coil count, and hypercoiling status.

Results: Oligohydramnios occurred in 24 (19.5%) pregnancies, and hypercoiling was present in 32 (26.0%) cases. For fetal distress, coil count remained independently associated with lower odds in the multivariable model (adjusted OR: 0.524, 95% CI: 0.287-0.881; p=0.023), though hypercoiling itself showed no association. For oligohydramnios, hypercoiling remained independently associated with higher odds (adjusted OR: 3.18, 95% CI: 1.12-9.08; p=0.0286).

Conclusions: We found that hypercoiling was independently associated with oligohydramnios, while higher coil count was independently associated with lower odds of fetal distress. These findings suggest that different methods of assessing cord coiling may capture distinct pathophysiologic mechanisms.

## Introduction

The umbilical cord is a remarkable structure. It must maintain adequate fetoplacental perfusion throughout pregnancy while simultaneously accommodating fetal movement, maternal positional changes, and uterine contractions. Its characteristic helical configuration -- first systematically described by Edmonds in 1954 -- has long been thought to serve a protective function, increasing tensile strength and reducing the risk of kinking or compression under mechanical stress [1,2].

Over the past several decades, numerous studies have attempted to link abnormal cord coiling with adverse pregnancy outcomes. Investigators have reported associations with intrauterine growth restriction, abnormal intrapartum fetal heart rate patterns, non-reassuring fetal status, and increased rates of operative delivery [3-7]. The biological rationale is compelling: if the helical structure truly protects vascular flow, then deviations from normal coiling -- whether too much or too little -- might compromise cord function under stress.

Yet despite this substantial body of work, the antenatal evidence remains frustratingly inconsistent [7-10]. Reviews by de Laat et al., Sebire, and Khong have all highlighted significant heterogeneity across studies, particularly in how “hypercoiling” and “hypocoiling” are defined [7,9,10]. Some investigators use percentile-based thresholds -- typically the 90th or 10th percentile for gestational age -- while others employ fixed cutoffs such as >0.3 coils/cm or <0.1 coils/cm. These different approaches inevitably produce different prevalence estimates and, potentially, different risk associations. The lack of standardization makes it difficult to synthesize findings or translate them into clinical practice.

There is also considerable outcome heterogeneity. The term “fetal distress,” for instance, encompasses a range of clinical scenarios and may reflect varying interpretations of cardiotocography (CTG) findings, different institutional thresholds for intervention, and subjective clinical judgment rather than a single objective physiologic state [11,12]. This imprecision complicates efforts to establish clear associations.

Oligohydramnios -- defined as reduced amniotic fluid volume for gestational age -- presents a somewhat different challenge. It is objectively measurable and clearly associated with adverse neonatal outcomes [13]. The condition often coexists with placental insufficiency, which raises an interesting question: could abnormal cord coiling and oligohydramnios share common upstream causes? [14-16] Several recent studies have suggested a relationship between the two, though no definitive causal mechanism has been established [14].

This study aimed to evaluate the clinical significance of umbilical cord hypercoiling detected during antenatal ultrasonography. The primary objective was to examine whether hypercoiling, defined using a fixed coil-density threshold, is associated with fetal distress at delivery after adjustment for relevant confounding variables. The secondary objective was to assess whether the same antenatal finding is associated with oligohydramnios, using a separate multivariable model.

## Materials and methods

Study design and setting

This prospective observational cohort study was conducted in the Department of Obstetrics and Gynecology at North Eastern Indira Gandhi Regional Institute of Health and Medical Sciences (NEIGRIHMS), Shillong, India, from January 2021 to July 2022. Ethical approval was obtained from the Institutional Ethics Committee, NEIGRIHMS (NEIGR/IEC/M14/T18/2021). Pregnant women meeting the eligibility criteria were enrolled at the time of second-trimester antenatal ultrasonography and followed until delivery.

Participants

Women with singleton pregnancies undergoing antenatal ultrasonography between 14 and 22 weeks of gestation were eligible for inclusion. Pregnancies with major obstetric or fetal conditions likely to independently affect cord morphology or perinatal outcome were excluded, including high-risk pregnancies, placental or umbilical cord anomalies, malpresentation, previous cesarean section, and other clinically significant complications as per protocol. Only participants with complete ultrasonographic cord assessment and delivery outcome data were included in the final analysis.

Ultrasonographic assessment of umbilical cord coiling

Umbilical cord coiling was assessed during second-trimester obstetric ultrasonography. A free loop of the umbilical cord was identified and visualized in the longitudinal plane, avoiding the fetal and placental insertion sites wherever feasible. The number of complete coils was counted over a measured cord segment using electronic calipers on a frozen image. Hypercoiling was defined using a predefined coil-density threshold of >0.3 coils/cm, equivalent to >3 coils per 10 cm, in keeping with fixed-threshold approaches described previously. For analysis, participants were classified as hypercoiling present or hypercoiling absent.

Outcomes

The outcomes of interest were oligohydramnios and fetal distress at delivery, both coded as binary variables (present/absent). Oligohydramnios was recorded from antenatal or intrapartum clinical documentation. Oligohydramnios was defined by radiological criteria of amniotic fluid index or a maximum vertical pocket. Fetal distress was defined according to the treating obstetrician’s clinical diagnosis based on fetal heart rate of less than 110 bpm or more than 140bpm, decreased fetal movement, presence of meconium-stained amniotic fluid, and poor doppler flow, if available. 

Statistical analysis

Continuous variables were summarized using mean and standard deviation or median and interquartile range, as appropriate, and categorical variables were expressed as frequency and percentage. Binary logistic regression was used to assess the association of antenatally detected hypercoiling with oligohydramnios and fetal distress. Separate models were fitted for each outcome. Crude ORs with 95% CIs were calculated. As an exploratory analysis, the number of coils was also entered as a continuous predictor, both alone and in combination with hypercoiling status. Model performance was assessed using the Hosmer-Lemeshow goodness-of-fit test, Nagelkerke’s pseudo-R², and the area under the receiver operating characteristic curve (AUC). A two-sided p-value <0.05 was considered statistically significant.

## Results

Association between hypercoiling and oligohydramnios

Oligohydramnios was more frequent among pregnancies with hypercoiling than among those without hypercoiling (12/32, 37.5% vs 12/91, 13.2%). On logistic regression, hypercoiling was significantly associated with increased odds of oligohydramnios (OR: 3.95, 95% CI: 1.55-10.10; p=0.004). The model demonstrated modest discrimination (AUC: 0.649) and explained approximately 10.2% of the variance in oligohydramnios (Nagelkerke R²=0.102).

When the number of coils was analyzed as a continuous predictor, the association showed a positive trend but did not reach statistical significance (OR: 1.36 per additional coil, 95% CI: 0.99-1.88; p=0.061). In the exploratory model, including both hypercoiling status and number of coils, hypercoiling remained significantly associated with oligohydramnios (OR: 3.11, 95% CI: 1.12-8.62; p=0.029), whereas the number of coils was not independently associated (OR: 1.19, 95% CI: 0.88-1.62; p=0.260). The combined model showed acceptable calibration (Hosmer-Lemeshow p=0.656), with modest discrimination (AUC: 0.716) and low explanatory power (Nagelkerke R²=0.120).

Association between hypercoiling and fetal distress

The proportion of fetal distress was similar in the hypercoiling and non-hypercoiling groups (6/32, 18.8% vs 18/91, 19.8%). Hypercoiling was not associated with fetal distress on logistic regression (OR: 0.94, 95% CI: 0.34-2.61; p=0.899). The model demonstrated no meaningful discriminatory ability (AUC: 0.506) and negligible explanatory value (Nagelkerke R²=0.0002).

**Figure 1:**
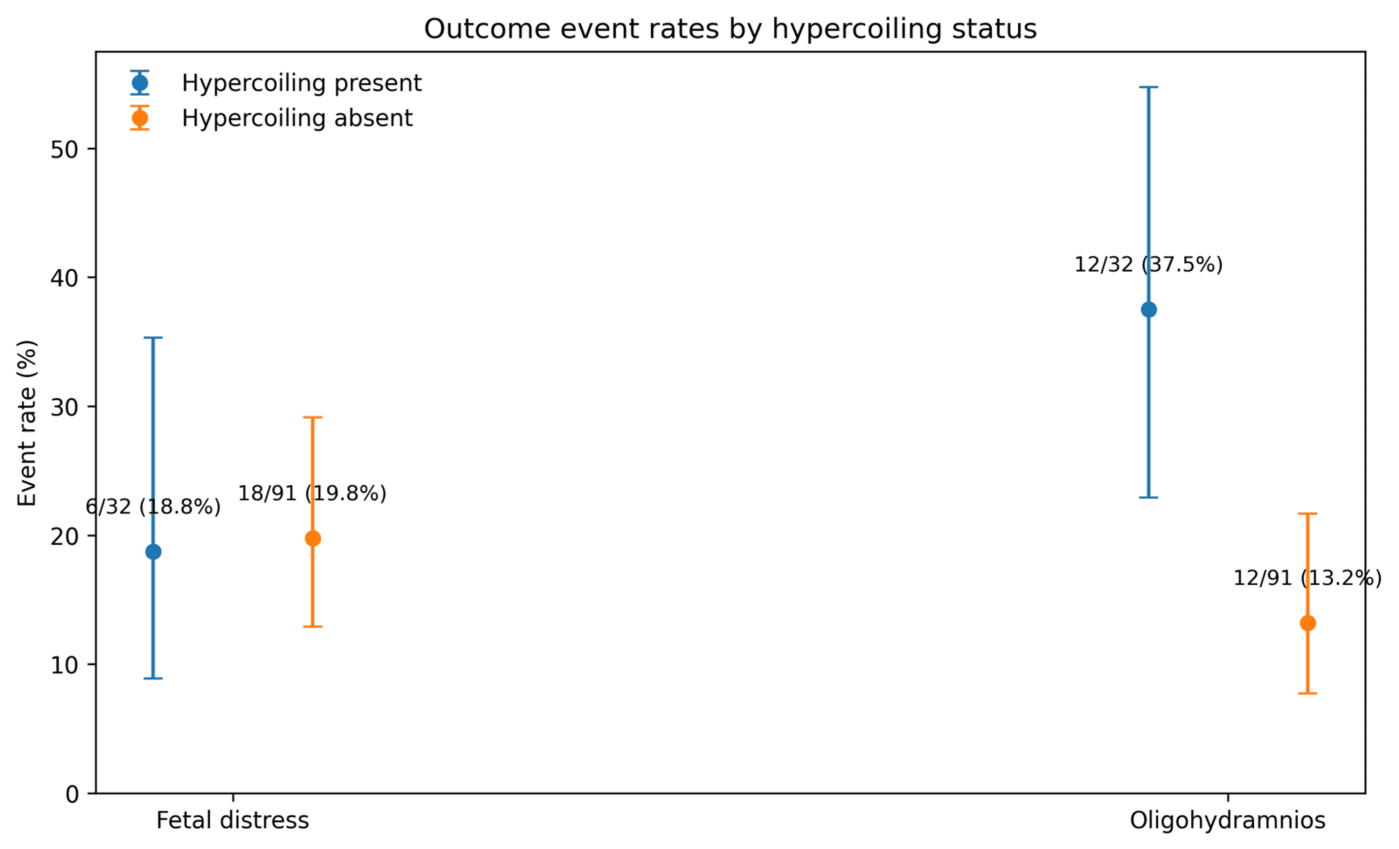
Event rates of fetal distress and oligohydramnios by umbilical cord hypercoiling status Event-rate dot plot comparing the proportion of fetal distress and oligohydramnios among pregnancies with and without umbilical cord hypercoiling. Dots represent observed event rates, and error bars represent 95% confidence intervals. Data labels show the number of events over the total number of cases in each group: fetal distress, 6/32 (18.8%) in the hypercoiling group vs 18/91 (19.8%) in the non-hypercoiling group; oligohydramnios, 12/32 (37.5%) vs 12/91 (13.2%), respectively. The figure was generated using Python with Matplotlib.

When the number of coils was examined as a continuous predictor, a higher coil count was associated with lower odds of fetal distress (OR: 0.61 per additional coil, 95% CI: 0.38-0.98; p=0.040). In the exploratory model, including both hypercoiling and number of coils, hypercoiling remained non-significant (OR: 1.73, 95% CI: 0.54-5.53; p=0.356), whereas number of coils retained an inverse association with fetal distress (OR: 0.56, 95% CI: 0.33-0.94; p=0.027). This model showed acceptable fit (Hosmer-Lemeshow p=0.784), modest discrimination (AUC: 0.637), and limited explanatory power (Nagelkerke R²=0.077). Potential collinearity between coil count and hypercoiling was not formally assessed. Figure 1 shows the event-rate dot plot for both fetal distress and oligohydramnios, with percentages. Overall, antenatally detected ultrasonographic hypercoiling was associated with increased odds of oligohydramnios but not fetal distress in this cohort. The continuous number of coils showed a different pattern, with a higher coil count being inversely associated with fetal distress in exploratory analysis.

## Discussion

In this observational cohort of 123 singleton pregnancies, we found that umbilical cord hypercoiling identified on antenatal ultrasonography was independently associated with oligohydramnios at delivery, with an adjusted OR of 3.18. Interestingly, the binary hypercoiling classification showed no association with fetal distress. Instead, it was the continuous measure -- absolute coil count -- that remained protective, with each additional coil reducing the odds of fetal distress by roughly half in the adjusted model. This divergence is striking and suggests that how we conceptualize and measure cord coiling may actually matter quite a bit.

The helical structure of the umbilical cord has fascinated obstetricians since Edmonds’ early descriptions in the 1950s [1]. Lacro et al. later proposed that this coiling serves a mechanical function, preventing kinking and maintaining vascular patency during fetal movement and uterine contractions [2]. Since then, a substantial literature has accumulated, linking abnormal coiling -- both hypercoiling and hypocoiling -- to various adverse outcomes, including growth restriction, abnormal heart rate patterns, and operative deliveries [3-7]. Yet despite decades of research, the antenatal associations remain surprisingly inconsistent across studies [7-10].

Part of the problem is definitional. Some investigators define hypercoiling as coil density above the 90th percentile for gestational age, while others use fixed cutoffs such as the 0.3 coils/cm threshold we employed here [7,8]. These different approaches produce different prevalence estimates and potentially different effect sizes, making it difficult to synthesize findings across studies. De Laat et al., Sebire, and Khong have all emphasized this methodological heterogeneity in their reviews [7,9,10].

Our finding that hypercoiling is associated with oligohydramnios is consistent with recent work by Kalluru et al., who reported similar associations in their cohort [14]. The biological mechanism underlying this relationship is not entirely clear. One possibility is that both conditions reflect a common upstream problem -- perhaps placental insufficiency or chronic hypoxemia -- that affects both amniotic fluid dynamics and cord morphogenesis. Another possibility is that reduced fetal movement, which can decrease amniotic fluid through reduced swallowing and urination, might also influence cord coiling during the critical first-trimester window when this helical pattern is established [15,16]. Our cross-sectional outcome assessment does not allow us to determine temporal sequence or causality. We also cannot rule out the possibility that oligohydramnios simply makes the cord easier to visualize on ultrasound, leading to more accurate coiling assessment, though this seems less likely given that we measured coiling in the second trimester when oligohydramnios is uncommon.

The protective effect of higher coil count against fetal distress is perhaps more intuitive. If the coiled structure really does buffer the cord against compression during labor, then more coils should logically provide better protection. This aligns with earlier work by Strong et al., who developed the umbilical coiling index and showed that both extremes -- very high and very low coiling -- were associated with adverse outcomes [3,4]. Rana et al. and Chitra et al. reported similar findings regarding abnormal fetal heart patterns [5,11]. What is particularly interesting in our data is that the continuous coil count measure remained predictive even when the categorical hypercoiling classification did not. This raises an important question: are we using the right cutoffs? A cord with moderately elevated coil density might function perfectly well during labor, whereas a cord with very few coils might be vulnerable to compression. The absolute number of coils -- which integrates both density and length -- may capture functional reserve better than a simple binary threshold.

We should be clear about the limitations of this work. First, this is an observational study, so we cannot establish causation. The associations we observed might reflect unmeasured confounding. For example, an underlying placental problem could independently increase both hypercoiling and oligohydramnios risk without one causing the other. Second, our definition of “fetal distress” relied on clinical documentation rather than standardized CTG interpretation criteria. The term itself is somewhat imprecise -- current guidelines favor more specific descriptors such as “non-reassuring fetal status” or particular CTG pattern classifications [17,18]. This outcome heterogeneity may have reduced our power to detect true associations. Third, our sample size of 123, while adequate for detecting moderate effects, limited our ability to perform subgroup analyses or detect smaller effects. Fourth, we did not systematically record cord length, which obviously influences absolute coil count and might independently affect outcomes. Fifth, our exclusion criteria, which removed high-risk pregnancies, placental anomalies, and prior cesarean sections, may limit generalizability. Finally, we did not examine the cords after delivery or perform histopathological analysis, which could have provided mechanistic insights.

On the positive side, we used a prespecified definition of hypercoiling, which improves reproducibility. We performed separate multivariable models for each outcome with appropriate covariate adjustment. And our cohort came from a single center with standardized ultrasonography protocols, reducing measurement variability. Finally, our findings need replication in other populations. This was a tertiary center study of singleton pregnancies with exclusion of high-risk conditions. Whether the same associations hold in multiple gestations, in pregnancies with fetal anomalies, or in lower-resource settings is uncertain. Future research should focus on prospective multicenter studies with standardized definitions and outcomes, longitudinal studies to clarify temporal relationships, mechanistic investigations to understand the underlying biology, and ultimately, randomized trials to test whether interventions can improve outcomes. While further validation is needed, our findings support the potential value of systematic cord coiling assessment in comprehensive antenatal care. With better understanding and standardized approaches, we may be able to reduce morbidity and mortality associated with cord abnormalities.

## Conclusions

We found that umbilical cord hypercoiling identified on antenatal ultrasonography is independently associated with oligohydramnios at delivery, with an adjusted OR of 3.18. We also found that a higher absolute coil count is independently associated with reduced odds of fetal distress, with an adjusted OR of 0.524. Importantly, these two measures -- binary hypercoiling classification and continuous coil count -- appear to capture different aspects of cord pathophysiology. The association between hypercoiling and oligohydramnios is biologically plausible, possibly reflecting shared underlying factors such as placental insufficiency or altered fetal activity. However, our cross-sectional design prevents us from establishing causality or temporal sequence. The protective effect of higher coil count against fetal distress supports the hypothesis that cord coiling provides mechanical protection during labor. Umbilical cord abnormalities remain an important contributor to pregnancy complications and adverse perinatal outcomes. Our results suggest that incorporating quantitative cord coiling parameters into antenatal risk assessment protocols could help identify at-risk pregnancies earlier and guide surveillance strategies.
